# Efficacy and Safety of Beta-Tricalcium Phosphate/Polylactic-Co-Glycolic Acid for Implantation of Bone Defects

**DOI:** 10.7759/cureus.43597

**Published:** 2023-08-16

**Authors:** Soichiro Tokeshi, Taisuke Fukawa, Eichi Itadera, Tsutomu Akazawa, Takayuki Fujiyoshi, Masashi Takaso, Koichi Nakagawa, Tomonori Yamauchi, Naoki Osada, Seiji Ohtori

**Affiliations:** 1 Department of Orthopaedic Surgery, Graduate School of Medicine, Chiba University, Chiba, JPN; 2 Orthopaedics, Narita Red Cross Hospital, Chiba, JPN; 3 Department of Orthopaedic Surgery, St. Marianna University, Kawasaki-City, JPN; 4 Orthopaedics, Kimitsu Chuo Hospital, Kisasrazu, JPN; 5 Orthopaedic Surgery, Kitasato University, Sagamihara, JPN; 6 Department of Orthopaedic Surgery, Toho University Sakura Medical Center, Sakura, JPN; 7 Orthodontics, Asahi General Hospital, Asahi, JPN; 8 Orthopaedics, ORTHOReBIRTH Co., Yokohama, JPN; 9 Orthopaedics, Chiba University Hospital, Chiba, JPN

**Keywords:** osteoblasts, absorbable implants, glycolic acid, osteogenesis, beta-tricalcium phosphate, bone graft substitutes

## Abstract

Introduction: Bone defects are often observed after surgery for fractures and bone tumors. Their treatment is technically difficult and sometimes results in negative clinical and economic outcomes. To repair bone defects, a bone graft is implanted by selecting a transplant material from an autologous or artificial bone. Each method has its advantages and disadvantages. Compared to the gold standard of autologous bone graft, bone graft substitutes are not limited by the amount of harvested graft and avoid complications at the donor site. ORB-03 is a new cotton-like bone graft substitute composed of beta-tricalcium phosphate (β-TCP) and a bioabsorbable polymer, polylactic-co-glycolic acid (PLGA). ORB-03 is easy to mold and can fill various bone defect shapes, and its three-dimensional microfiber scaffold can enhance the differentiation of osteoblasts and promote osteogenesis. We investigated the efficacy, ease of handling, and safety of ORB-03 as a bone graft substitute. A multicenter, open-label, single-group study was conducted at six institutions.

Methods: Between July 2018 and August 2019, 60 patients with bone defects caused by fracture, benign tumors, or an iliac donor site from bone harvesting were enrolled in this study; 54 patients were finally included for the safety analysis and 48 patients for the image analysis. During surgery, ORB-03 was mixed with the patient's blood and molded into a bone defect. To evaluate the efficacy of ORB-03, radiography and computed tomography (CT) were performed at intervals until 24 weeks after surgery.

Results: The effective rate and its accurate bilateral 95% confidence interval (CI) were calculated based on the efficacy criteria at 24 weeks postoperatively. The ease with which ORB-03 could be handled in surgery was evaluated. Adverse events that occurred after surgery were evaluated, and those associated with ORB-03 were examined. Bone fusion was good in all cases, and the radiography and CT effective rates were 100.0% and 91.5%, respectively. Handling was easy in all cases. There were four adverse events, none of which were clinically problematic.

Conclusions: ORB-03 was found to be easy to handle, safe, and effective as a bone graft substitute for bone defects.

## Introduction

Bone defects are often observed after surgery for fractures and bone tumors. The treatment of bone defects is difficult because of postoperative complications such as infection and pseudoarthrosis, and their management can be clinically and economically expensive [1]. To repair a bone defect, a bone graft is implanted by selecting a transplant material from autologous, allogeneic, or artificial bone. Each method has advantages and disadvantages. Autologous bone grafts, especially iliac crest bone grafts, are the gold standard because of their osteogenic capability and safety; however, there is a limit to the amount harvested, and there are various donor site problems, such as postoperative pain and sensory disturbance at the donor site [2,3]. Allografts are used as alternatives to autografts and are free from the donor site problems that occur with the use of autografts; however, facilities that can use them are limited in Japan [4,5]. Although bone graft substitutes differ in material composition, internal structure, and strength, they have the advantages of being free from volume limitations and donor site problems and are easy to use.

Hydroxyapatite (HA) and beta-tricalcium phosphate (β-TCP) are often used; however, in recent years, new bone graft substitutes have been developed and are widely used. The porous HA/collagen complex (HAp/Col) has a structure and composition similar to those of natural bone [3,6,7]. Demineralized bone matrix (DBM) is a supplementary material with bone-inducing ability [5,6].

Bone graft substitutes have also been improved to conform more closely to the shape of defects. It has been reported that the bone fusion rate can be increased by changing the porosity and nanoscale shape. Soft bone graft substitutes that do not leak after surgery and that conform to bone defects have also been developed [7,8]. ORB-03 is a new cotton-like artificial bone made of 60-80% β-TCP and 20-40% bioabsorbable polymer consisting of polylactic-co-glycolic acid (PLGA: copolymerization ratio 85:15), which is easy to mix with autologous bone. ORB-03 can be easily molded into various shapes and can fill various bone defects; it is marketed as ReBOSISS-J in Japan. While recent clinical research has focused on materials with superior bone healing properties, such as the study of bone morphogenetic proteins (BMPs) to target early bone healing, few studies have addressed the ease of handling artificial bones, such as shape and leakage [9]. While recent clinical research has focused on materials with superior bone healing properties, such as the study of BMPs to target early bone healing, few studies have addressed the ease of handling artificial bones, such as shape and leakage [9]. The purpose of this study was to assess the efficacy and safety of ORB-03 for treating bone defects and to evaluate its ease of handling during surgery.

## Materials and methods

This multicenter, open-label, single-group study was conducted at six institutions. The institutional review board approved the study of each institution. Informed consent was obtained from all patients. Between July 2018 and August 2019, 60 patients who required bone substitute implantation for bone defects caused by fractures, benign tumors, or in the iliac donor site from bone harvesting were enrolled in this study. The inclusion and exclusion criteria are presented in Table 1. All participants provided written informed consent before inclusion in the study. All protocols involving humans were approved by all involved institutions and performed in accordance with the ethical standards laid down in the 1964 Declaration of Helsinki and its later amendments.

**Table 1:** Inclusion and exclusion criteria. PLGA: poly lactic-co-glycolic acid.

Inclusion criteria: patients who met all the following criteria were included in this trial
Patients >20 years of age
The volume of the bone defect was ≤30 mL
Patients whose general condition was sufficiently stable for surgery
Patients who provided first-person informed consent
Exclusion criteria: patients who met any of the following criteria were excluded
1. Patients with any of the following conditions currently or in the past:
Severe osteomyelitis
Severe diabetes (poor control with drug therapy)
Chronic kidney failure
Abnormal hormonal metabolism
2. Patients who were administered the following drugs within three months prior to implantation:
Parathyroid hormone
Humanized antisclerostin monoclonal antibody
Steroid agents (excluding external preparations, eye drops, and nasal drops)
Immunosuppressive agents
3. Patients with the following conditions:
Open fractures intra-articular fracture requiring autologous bone graft
Transplantation sites with severe vascular insufficiency or neurological disorders
Pathological fractures except for those caused by osteoporosis
Allergic responses to β-tricalcium phosphate or PLGA
Pregnant women

Surgical procedure

**Figure 1:**
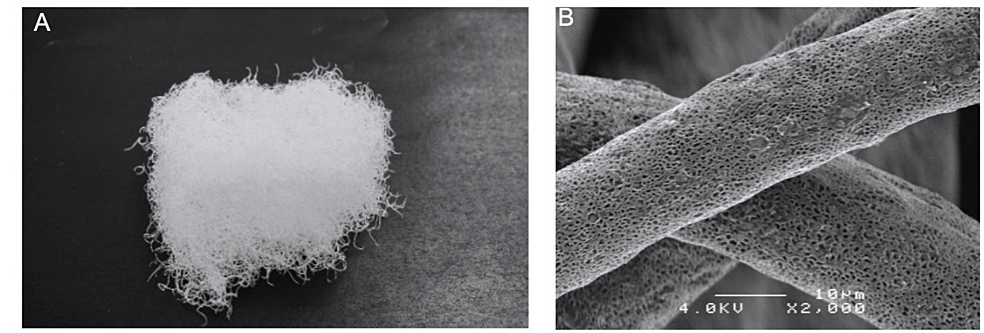
Macroscopic appearance of ORB-03 (A) and at 2000× magnification (B).

The surgery was performed by orthopedic surgeons who had been trained in advance to manage ORB-03. During surgery, 1.0 mL of ORB-03 was mixed with approximately 4.0 mL of the patient's blood to make it easier to calculate the amount filled and molded into the bone defect (Figure 1). The amount of ORB-03 implanted was limited to a maximum of 6.0 mL, and the filling amount was accurately recorded from the remaining amount implanted. In the case of a fracture, internal fixation was performed with a plate and screw so that the filled ORB-03 was not loaded directly. In the bone tumor cases, the lesion was completely curetted, and ORB-03 filled the cavity. The mixed-use of other bone graft substitutes or agents for the same bone defect, the use of other bone graft substitutes for another site in the same patient, and biophysical stimulation therapy for the filling site (such as medium-frequency, low-frequency, ultrasonic, microwave, or magnetic therapy) were prohibited to accurately evaluate ORB-03.

Radiographic assessment

To evaluate the efficacy of ORB-03, radiographs in two directions were obtained before surgery and at four, eight, 12, 18, and 24 weeks after surgery. According to previous reports, the status of bone fusion was scored on a scale of four points by three radiologists who were not involved in the treatment (Table 2) [3,7]. We defined three points as effective and four points as highly effective, and the effective rate was calculated as the number of patients with a score of three or more points per patient. Computed tomography (CT) images were obtained 24 weeks after surgery to evaluate the status of bone fusion based on the scoring criteria in Table 2.

**Table 2:** Radiographic criteria to evaluate the efficacy of grafted bone fusion.

Evaluation		Score	
A	Evaluation of marginal zone (osteointegration, radiolucent line)	2	All-around osteointegration
		1	Partial osteointegration
		0	All-around radiolucent line
B	Bone regeneration (degradation and replacement of the implant)	2	Complete replacement by regenerated bone
		1	Partial replacement by regenerated bone
		0	No bone regeneration or no degradation of implants
A total score of 3 or more of A: marginal zone + B: bone regeneration was defined as effective			

Ease of handling

The ease with which ORB-03 could be handled during surgery was evaluated based on (1) moldability according to the filling part of the filling material, (2) ease of embedding the filling part, and (3) leakage of ORB-03 from the filling part during surgery. The orthopedic surgeon evaluated each item on a two-point scale (good or bad) before closing the wound. We defined "leakage" as the presence of ORB-03 outside of the donor site at the time of wound closure.

Safety and adverse events

Blood tests and medical examinations were performed before surgery, immediately after surgery, and at four, eight, 12, 18, and 24 weeks after surgery. Adverse events that occurred after surgery were evaluated on a three-point scale (mild, moderate, or severe), and those associated with ORB-03 were examined. Any symptoms that occurred before the study were excluded from the adverse events.

Statistical analysis

The effective rate and its accurate bilateral 95% confidence interval (CI) were calculated based on the efficacy criteria at 24 weeks postoperatively. Based on previous reports, we judged that ORB-03 was effective when the accurate bilateral 95% CI lower limit of the effective rate was ≥60%, and we hypothesized that the effective rate of ORB-03 would be approximately 80% [3,7]. Under these conditions, the number of cases required to ensure an accurate bilateral 95% CI and a power of at least 80% was calculated to be 48 cases per group. We finally set the target number of cases per group at 54, estimating a 10% incidence of cases excluded from analysis owing to discontinuation or other reasons. SAS version 9.4 (SAS Institute Co., Tokyo, Japan) was used for statistical analysis.

## Results

**Figure 2:**
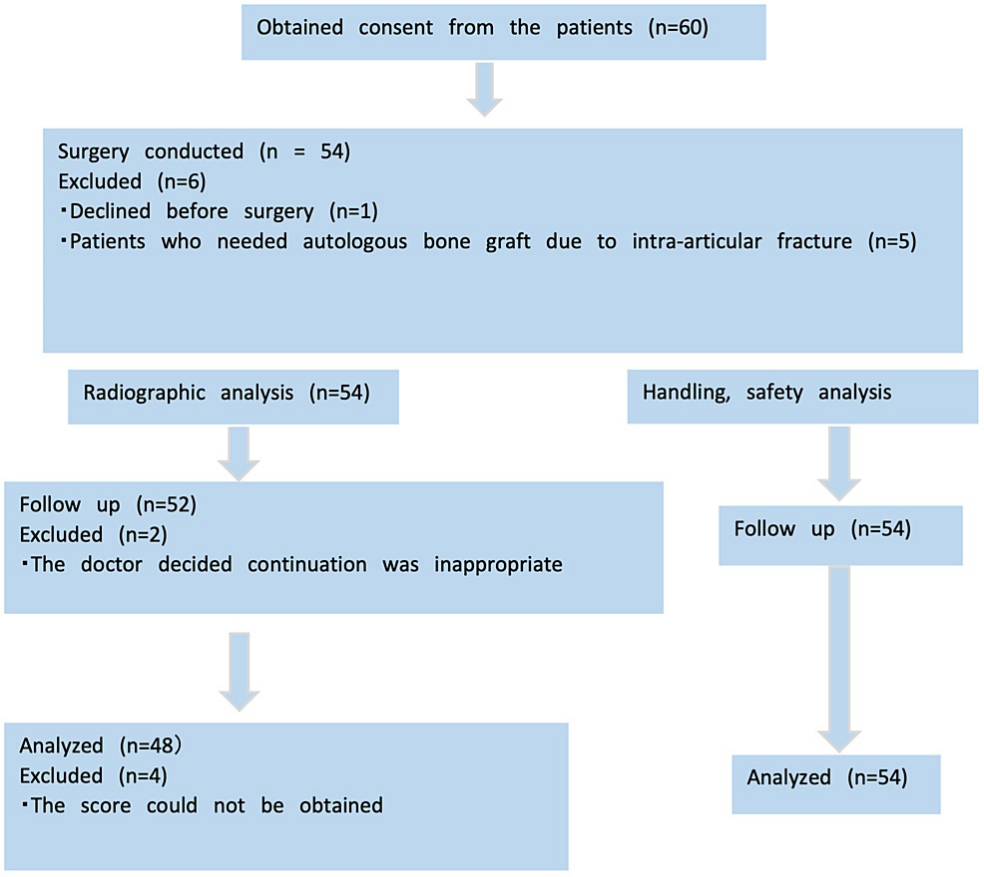
Enrollment of study participants.

Of the 60 enrolled patients, 59 underwent surgery. One patient was excluded before the start of the study, and five patients with intra-articular fractures requiring autologous bone grafting were excluded. Of the 54 patients in whom ORB-03 was implanted, two were discontinued, and four could not be followed up postoperatively. Finally, 48 patients were included in the image analysis, and 54 patients were included in the safety analysis (Figure 2). ORB-03 was used most frequently for fractures (29 cases, 60.4%), of which distal radius fractures were the most common (24/29 cases, 83.0%) (Table 3). The average volume of implanted blood contained in ORB-03 was 4.12 ± 3.60 mL, with 23 cases (47.9%) having an implanted amount of <3 mL, 16 cases with 3 mL to <10 mL (33.3%), and nine cases (18.8%) with ≧10 mL. There were 32 patients (66.7%) who required internal fixation of the fractures (Table 4).

**Table 3:** The number of ORB-03 supplemented parts.

Bone defect caused by	Location of the defect	
Fracture from trauma	Radius	24
	Humerus	2
	Tibia	1
	Calcaneus	2
Benign bone tumor	Metacarpal bone	1
	Proximal phalange	2
	Tibia	1
Harvesting of autograft	Ilium	15
Total	48	

**Table 4:** Characteristics of enrolled patients.

Index	
Sex (male/female)	14/34
Mean age (years)	62.2 ± 15.3
Mean amount of implanted blood containing ORB-03 (mL)	4.12 ± 3.60
Internal fixation conducted	32
No internal fixation	16

Radiographic assessment

The effective rate and 95% CI bilaterally were calculated based on the radiographic evaluation at four, eight, 12, 18, and 24 weeks after surgery. The scores based on radiographic evaluation at each postoperative time point improved over time. Bone fusion was good in all 48 cases, and the effective rate was 100.0% (48/48 cases, bilateral 95% CI 92.6-100.0) (Table 5). The highly effective rate was 87.5% (42/48 cases, bilateral 95% CI 74.8-95.3). Forty-eight patients underwent CT examinations 24 weeks after surgery. One patient was excluded because the involved site could not be identified. The effective rate was 91.5% at 24 weeks after surgery (43/47 cases, bilateral 95% CI 79.6-97.6).

**Table 5:** Radiographic effective (total score ≥3) rate at each time point after surgery.

Postoperative timing (weeks)	Patients	Effective patients	Effective rate (%)	95% confidence interval	
				Low	High
4	48	1	2.1	0.1	11.1
8	47	15	31.9	19.1	47.1
12	48	39	81.3	67.4	91.1
18	48	46	95.8	85.7	99.5
24	48	48	100	92.6	100

Ease of handling

The ease of handling was evaluated as easy in all cases, and there were no cases of ORB-03 leakage during surgery.

Safety and adverse events

No severe adverse events, such as implantation site infection, bone fusion failure, or refracture, were observed. Four mild adverse events were associated with ORB-03: one each (1/54, 1.9%) of surgical site discomfort, itching, elevated aspartate aminotransferase (AST), and elevated alanine aminotransferase (ALT), none of which were clinically problematic. Discomfort at the surgical site occurred postoperatively and did not resolve during the observation period; however, the symptoms were mild and not of clinical concern. Itching at the surgical site appeared postoperatively, and the patient was quickly cured with topical medication.

Case presentations

Case 1

**Figure 3:**
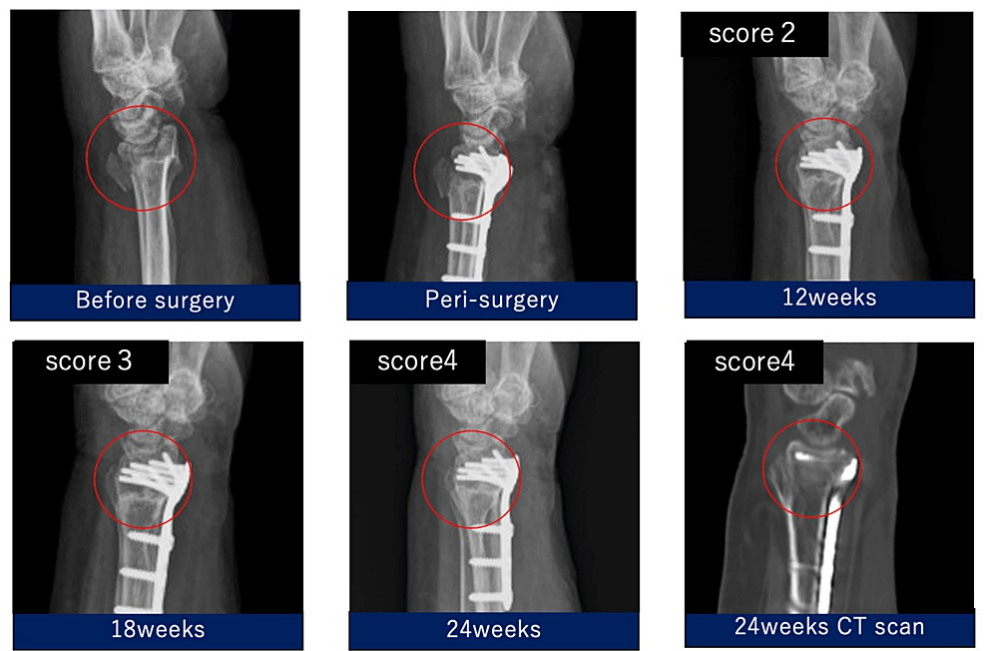
Radiographic evaluation of implanted ORB-03 for distal radius fracture. The circled area was filled with blood containing ORB-03 for the distal radius fracture and imaged preoperatively and at 12, 18, and 24 weeks after surgery.

Blood containing ORB-03 (1.95 mL) was used to fill a distal radius fracture defect in a 76-year-old woman. The bone fusion score was two points at 12 weeks, which improved over time. Finally, at 24 weeks, the bone fusion score was four points, confirming fusion (Figure 3).

Case 2

**Figure 4:**
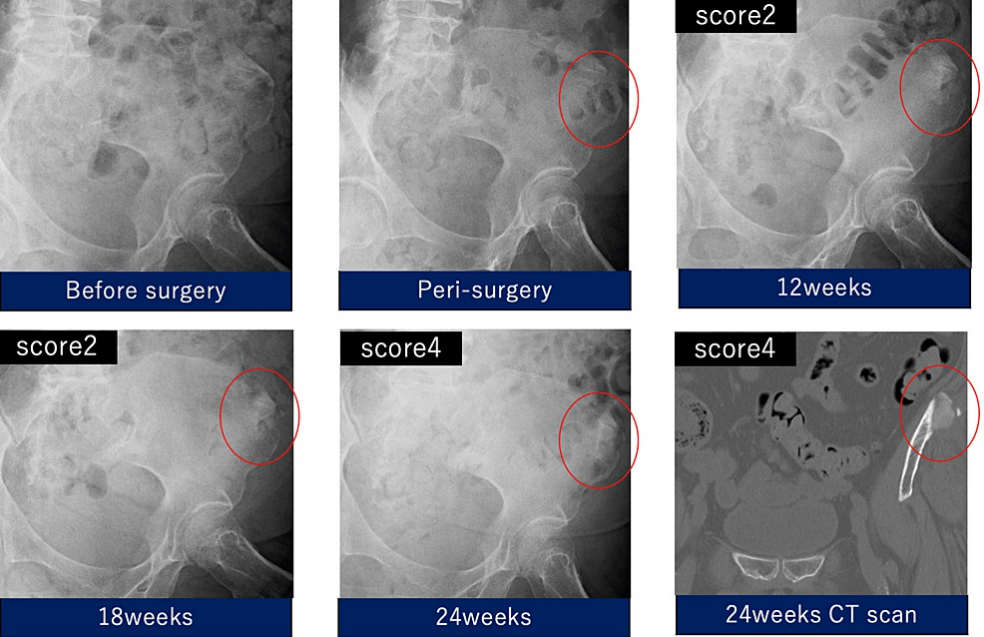
Radiographic evaluation of implanted ORB-03 as an iliac bone graft. The circled area was filled with blood containing ORB-03 for the harvested site of ilium and imaged preoperatively and at 12, 18, and 24 weeks after surgery.

Blood containing ORB-03 (10.0 mL) was grafted onto the harvested site of the ilium of a 72-year-old female patient. Partial osteointegration and replacement with the regenerated bone occurred within 12 weeks. At 24 weeks, the total bone fusion score was four points, confirming fusion (Figure 4).

## Discussion

Various types of bone grafts, including autologous grafts, allografts, and bone graft substitutes, have been used to treat bone defects. Compared to the gold standard of autologous bone graft, bone graft substitutes are not limited by the amount of harvested graft and avoid complications at the donor site. Ideal bone graft substitutes should be osteoconductive, osteoinductive, biocompatible, bioresorbable, structurally similar to bone, easy to use, and cost-effective [10].

Generally used solid materials, such as porous HA and β-TCP, may require shaping depending on the shape of the bone defect and may leak after grafting [11]. Soft materials do not have mechanical strength but are easy to handle, and there is limited leakage after surgery. Because of ORB-03’s cotton-like texture and shape, the transplantation site was initially relatively weak. However, it was easy to mold and insert, with no leakage or loss of reduction during surgery. As a similar cotton-shaped bone graft substitute, ReBOSSIS®︎ (ORTHOReBIRTH Co., Kanagawa, Japan), composed of β-TCP, calcium carbonate, silicon, and PLGA, has been approved by the United States Food and Drug Agency (FDA). The silicon contained in ReBOSSIS®︎ is reported to enhance bone formation and stimulate osteogenic cells to mineralize the new bone [12]. ReBOSSIS®︎ has not been approved in Japan as a bone graft substitute [12]. Porous HAp/Col and DBM are soft bone graft substitutes similar to ORB-03 available in Japan. Refit® (HOYA Technosurgical Co., Tokyo, Japan) is a sponge-like bone graft substitute made of HAp/Col, which is osteoconductive and bioabsorbable and is easy to use to fill a bone defect with less leakage [3]. Refit® is similar to ORB-03 in terms of ease of handling because it is soft; however, the two graft substitutes differ in terms of their osteoconductive components (Refit®: HA, ORB-03: TCP) and the scaffold material used for osteoconduction (Refit®: type-1 atelocollagen fiber, ORB-03: PLGA fiber). Shinomiya et al. and Sotome et al. each evaluated bone fusion following the use of Refit®︎ for bone defects caused by fracture, benign tumors, or harvesting from iliac donor sites [3,7]. The bone fusion effective rate was evaluated using the same radiographic evaluation and scoring methods used in this study. The effective rate at 24 weeks after surgery was approximately 90% [3,7]. DBM is decalcified from allogeneic human bone tissue and is both osteoconductive and osteoinductive [5,6,13]. In contrast, ORB-03 is osteoconductive but not osteoinductive. DBM is frequently used in other countries, with some reports of good bone fusion in the spine and post-trauma cases [14,15]. In a report on the use of DBM for bone defects in periarticular fractures, Grafton®︎ (Osteotech, Eatontown, NJ, USA), which is made of DBM and glycerol carrier, and Orthoblast®︎ (Gensci, Irvine, CA, USA), which is made of DBM and poloxamer carrier, were implanted into bone defects, and bone healing was evaluated by radiographic examination at 12 months postoperatively. Bone healing occurred in 69% (9/13) of cases with Grafton®︎ and 100% (15/15) with Orthoblast®︎ [16].

Osferion® (Olympus Co., Tokyo, Japan), a highly purified β-TCP that is a hard bone graft substitute with the same components as ORB-03, is reported to have a bone fusion effective rate (score 3) of approximately 80% at 24 weeks after surgery [3,7]. In the current study, ORB-03’s effective rate at 24 weeks after surgery was 100% (48/48), and the highly effective rate (score 4) was 87.5% (42/48). Although not a direct comparison, ORB-03 was as effective as other soft bone graft substitutes and solid β-TCP bone graft substitutes with the same components as ORB-03.

One of the advantages of ORB-03 is that it can be absorbed and replaced by newly formed bones. HA is a ceramic artificial bone widely used as a bone graft material. It has mechanical strength but has the disadvantage of not being absorbed and replaced by new bone, creating a risk for postoperative fracture [17-19]. The β-TCP in ORB-03 is osteoconductive and is absorbed and replaced by newly formed bone. The PLGA in ORB-03 is a biocompatible polymer [17,18] with an ester bond site that is hydrolyzed in the body and converted to lactic and glycolic acids. It decomposes into water and carbon dioxide via the tricarboxylic acid (TCA) cycle and is then excreted [20].

The three-dimensional microfiber scaffolds of cotton-like bone graft substitutes efficiently promote osteogenesis. According to a previous report, the process of osteogenesis using ReBOSSIS® in vitro, observed using an electron microscope, revealed that cells spread along the fibers [21] and indicated that a three-dimensional microfiber scaffold can enhance the differentiation of osteoblasts [21]. The biodegradation of PLGA used in this study lasted approximately 120 days [22]. On the other hand, an animal experiment using the canine mandible revealed that most β-TCP is absorbed within 12 weeks [23]. Therefore, PLGA is absorbed more slowly than β-TCP, and this degradation time lag suggests that after β-TCP degradation, PLGA fibers allow osteogenic cells to invade easily. This could enhance efficacy by functioning as a scaffold for osteogenesis over a prolonged period. However, histological evaluation is necessary to determine whether ORB-03 remains at the graft site.

To the best of our knowledge, β-TCP does not cause serious adverse events; however, mild postoperative inflammation, graft infection, and mild elevation of hepatobiliary enzyme levels have been reported [3,7]. Refit®︎, another soft bone graft substitute, was associated with a transient foreign body reaction at the grafted site [3,7]. With DBM, ectopic ossification and tumor formation are possible [14]. DBM is osteoinductive; however, its osteoinductivity varies depending on the product because the sterilization process during manufacturing adversely affects it [13]. In this study, four adverse events were associated with ORB-03; however, the symptoms were mild and did not pose a clinical problem.

This study has several limitations. First, the number of patients was small, although the 59 patients in this study are comparable to those in previous reports on graft substitutes for bone defects [7,8,16-19,24,25]. Nevertheless, further research is needed to verify the results of this study. Second, the ease of handling was evaluated subjectively. We were unable to find studies that objectively evaluated the handling of bone grafts placed in bone defects. One objective measure of handling is the time from the opening of the package to molding. In this study, the subjective operability was good, but it was unclear how handling affected the clinical results. Third, 50% of cases were distal radius fractures. In previous reports, the percentage of upper extremity fractures repaired with artificial bone grafts was between 10% and 63%, but there were no reports of differences in bone healing by site [7,8,16-19,24]. In this study, the bone fusion score was good regardless of the grafted site, but further studies are necessary. Fourth, this study may have involved a highly selective inclusion of patients in good physical condition. The evaluation of bone fusion in this study was performed only using radiography and CT without histological analysis. The efficacy and safety of ORB-03 based on these evaluations were favorable; however, further clinical studies with more patients and histological evaluations are necessary. If the cotton-shaped structure is effective for bone healing, it may be possible to combine it with a carrier composed of a material that promotes bone healing (such as BMPs) in the future to further accelerate bone healing.

## Conclusions

 In this study, the efficacy and safety of ORB-03 for bone defects were confirmed, and excellent operability was demonstrated. Bone defects are challenging to treat and can have negative clinical effects. It is important to understand and utilize the advantages of artificial and autologous bone in the treatment of bone defects. Thus, ORB-03 can be used to treat various types of bone defects.
